# A novel approach to brain tumor detection using K-Means++, SGLDM, ResNet50, and synthetic data augmentation

**DOI:** 10.3389/fphys.2024.1342572

**Published:** 2024-07-15

**Authors:** Ponuku Sarah, Srigiri Krishnapriya, Saritha Saladi, Yepuganti Karuna, Durga Prasad Bavirisetti

**Affiliations:** ^1^ School of Electronics Engineering, Vellore Institute of Technology, Vellore, India; ^2^ School of Electronics Engineering, VIT-AP University, Amaravati, India; ^3^ Department of Computer Science, Norwegian University of Science and Technology, Trondheim, Norway

**Keywords:** K-means++ clustering, SGLDM, Resnet50, synthetic data augmentation, grad-CAM, deep learning

## Abstract

**Introduction:** Brain tumors are abnormal cell growths in the brain, posing significant treatment challenges. Accurate early detection using non-invasive methods is crucial for effective treatment. This research focuses on improving the early detection of brain tumors in MRI images through advanced deep-learning techniques. The primary goal is to identify the most effective deep-learning model for classifying brain tumors from MRI data, enhancing diagnostic accuracy and reliability.

**Methods:** The proposed method for brain tumor classification integrates segmentation using K-means++, feature extraction from the Spatial Gray Level Dependence Matrix (SGLDM), and classification with ResNet50, along with synthetic data augmentation to enhance model robustness. Segmentation isolates tumor regions, while SGLDM captures critical texture information. The ResNet50 model then classifies the tumors accurately. To further improve the interpretability of the classification results, Grad-CAM is employed, providing visual explanations by highlighting influential regions in the MRI images.

**Result:** In terms of accuracy, sensitivity, and specificity, the evaluation on the Br35H::BrainTumorDetection2020 dataset showed superior performance of the suggested method compared to existing state-of-the-art approaches. This indicates its effectiveness in achieving higher precision in identifying and classifying brain tumors from MRI data, showcasing advancements in diagnostic reliability and efficacy.

**Discussion:** The superior performance of the suggested method indicates its robustness in accurately classifying brain tumors from MRI images, achieving higher accuracy, sensitivity, and specificity compared to existing methods. The method's enhanced sensitivity ensures a greater detection rate of true positive cases, while its improved specificity reduces false positives, thereby optimizing clinical decision-making and patient care in neuro-oncology.

## 1 Introduction

The human brain is a highly intricate organ that performs a multitude of functions through the activity of billions of cells. An irregular proliferation of brain cells that occurs when the cells surrounding the brain begin to multiply uncontrollably is referred to as a brain tumor (Saba et al., 2020). Hence, the development of cell groups and the destruction of healthy cells result in abnormal brain activity or have an impact on normal function. The two main categories of brain tumors in humans are malignant and benign (Sharif et al., 2020). Benign tumors, which are generally considered non-malignant, non-cancerous, or non-proliferative, exhibit a less aggressive nature, have a moderate rate of growth, and cannot metastasize to other areas of the body, unlike their malignant counterparts. Among the most serious tumors are brain tumors (Amin et al., 2020). Brain tumors typically have complicated shapes and differ substantially in size, texture, and position. As a result, clinical data about tumors show significant spatial and structural heterogeneity between patients (Pereira et al., 2016). According to some experts, MRI is one of the best imaging methods for predicting tumor growth in both the detection and treatment phases (Vijh et al., 2020). The MRI is the most versatile imaging technology for depicting specific areas in the brain, such as tumors, as it normalizes the tissue contrast (Yazdan et al., 2022). The ability of MRI scans to provide a range of data, including the structure of the brain and anomalies within the brain tissues, makes them extremely important in the realm of medical image analysis (Arunkumar et al., 2020).

The exponential development of deep learning networks has made it possible for us to handle challenging jobs, even in the intricate field of medicine. One of the study fields that has generated the most interest recently is deep learning (Pavan et al., 2020) However, not everything in Deep learning is straightforward. Having a large enough amount of data to effectively train AI models for various tasks is one of the primary issues. The most important aspect of this restriction is how expensive or few study volunteers have access to images in the medical field (Mahmud et al., 2023). In addition, respondents must consent to be scanned, and they are free to decline even though their data is anonymized (Willemink et al., 2020). Delineating diseased areas is necessary for medical image segmentation. Segmenting brain tumors manually is a task that demands a lot of time and is susceptible to mistakes, especially when the tumors possess irregular shapes, sizes, and characteristics. Therefore, it is crucial to devise automated techniques for the segmentation of brain tumors that can enhance the precision and productivity of the model (Abdelaziz Ismael et al., 2020).

Deep learning techniques are thought to be effective for segmentation; convolutional neural network (CNN) (Amin et al., 2018) in particular is used for pattern identification. When compared to statistical techniques, such as support vector machines (SVM) (Shah et al., 2020) which rely on manually extracted features, these methods learn features in the form of a hierarchy. For the study of medical images, including pre-processing, segmentation, and classification, Deep CNN models are successfully used (Krishnapriya and Karuna, 2024). The latest research studies for the identification of brain tumors offered various classification and segmentation algorithms (Krishnapriya Srigiri and Karuna Yepuganti, 2023). A comprehensive analysis of prior research reveals that the overwhelming majority of inquiries encounter or neglect to consider the prevalent issues of overfitting and inadequately sized datasets (Liaqat et al., 2018). Huge hidden layers that extract noisy characteristics that impair the effectiveness of the classifier are one cause of the overfitting issue (Paul and Bandhyopadhyay, 2012).

Shree and Kumar (2018) utilized a probabilistic neural network (PNN) classifier for classifying normal and pathological brain MRIs based on GLCM-derived features. DCNNs are widely used for their exceptional performance but require significant computational resources. Various CNN models, including ResNet-50, DenseNet-201, Inception V3, and Google Net (Deepak et al., 2019), were employed to achieve high accuracy. Abd-Ellah et al. (2020) improved a deep CNN architecture for brain tumor detection, reaching 97.79% accuracy. Saxena et al. (2019) applied transfer learning approaches to Inception V3, ResNet-50, and VGG-16 models, achieving the best accuracy rate of 95%. Khan et al. (2020) used deep transfer learning methods to classify brain tumors from MRI scans, improving the VGG-16 model using the Brain Tumor Segmentation (BraTS) dataset and achieving an accuracy of 96.27%.

A recent study suggests using transfer learning networks to categorize brain cancers in MR images using pre-trained models from VGG16, VGG19, ResNet50, and DenseNet21, trained using four optimization strategies including Adadelta, Adam, RMSprop, and SGD (Maqsood et al., 2022). In the Figshare dataset, containing 3064 MR images from 233 patients with various brain tumors, ResNet50, optimized with Adadelta, achieved the highest classification performance at 99.02%.

Çinar and Yildirim (2020) suggested a technique that substituted the primary tumor region with an augmented tumor region obtained via image dilation as the region of interest (ROI). The authors then divided the enlarged tumor region into small, ring-shaped subregions using feature extraction techniques such as the intensity histogram, the gray level co-occurrence matrix (GLCM), and the bag-of-words (BoW) model. The results indicated a significant enhancement in classification accuracy for the intensity histogram, GLCM, and BoW model, increasing from 71.39% to 82.31%, 78.18%–84.75%, and 83.54%–88.19%, respectively (Krishnapriya S. and Karuna Y., 2023).

Anitha et al. (2022) applied deep learning techniques to classify lung diseases using chest X-ray (CXR) images. ResNet50 achieved 86.67% validation accuracy, while DenseNet outperformed with 98.33%. Comparative analysis favored DenseNet, highlighting its superior efficiency in lung disease classification. These findings emphasize the potential of deep learning architectures for improving early diagnosis of lung diseases.

Roy and Shoghi (2019) used a hybrid segmentation approach, combining fast k-means clustering, morphology, and level set techniques, for computer-aided tumor segmentation in T2-weighted MR images of patient-derived tumor xenografts. The method improved segmentation accuracy, resulting in an increased Dice score. Roy et al. (2017a) developed an iterative Level Set method for precise MRI brain tissue segmentation, encompassing normal tissues and abnormalities. The method generated a hierarchical structure for accurate segmentation, validated by evaluation metrics like accuracy and similarity index. Their work significantly contributes to enhancing precision in brain tissue segmentation within the field of medical image analysis.

Roy et al. (2017b) propose a novel computerized MRI brain binarization method, focusing on refining feature extraction and abnormality identification. The method addresses challenges, such as extensive black backgrounds and contrast variations, by utilizing mean, variance, standard deviation, and entropy for threshold determination, along with a non-gamut enhancement. Their approach, extensively tested on diverse MRI datasets, demonstrates superior accuracy and reduced errors compared to established methods. The comparative analysis highlights its effectiveness, marking a significant advancement in MRI preprocessing for enhanced brain imaging.

Gangopadhyay et al. (2022) developed MTSEU-Net, a groundbreaking architecture for fetal brain imaging that performs three tasks in a single framework: segmenting the fetal brain into seven components, predicting the brain type, and estimating gestational age. The model achieved a Jaccard similarity of 77% and a Dice score of 82%, demonstrating robust segmentation performance. It achieved impressive accuracies of 89% for predicting brain type and 0.83 weeks for estimating gestational age. This work represents a significant advancement in fetal brain imaging tasks. Roy et al. (2022) reviewed the integration of supervised machine learning (SML) in healthcare 4.0, emphasizing its potential across various sectors and the need for explainable AI. The insights provided serve as a valuable guide for researchers in academia and industry, shaping future discourse on SML in the healthcare and biomedical sectors.

Roy et al. (2017c) present an efficient method for computerized prediction and segmentation of multiple sclerosis (MS) lesions in brain MRI. The approach integrates adaptive background generation, global threshold-based binarization, and a three-phase level set for comprehensive lesion detection and segmentation. Notably, the method addresses spurious lesion generation and over-segmentation issues. Results demonstrate high accuracy with an average Kappa index of 94.88%, a Jacard index of 90.43%, a correct detection ratio of 92.60%, a false detection ratio of 2.55%, and a relative area error of 5.97%. This method successfully detects and accurately segments MS lesions in brain MRI. Furthermore, the method has the potential to improve clinical diagnosis and monitoring of MS progression. Additionally, it shows promise for enhancing treatment planning and evaluation in patients with multiple sclerosis.

**FIGURE 1:**
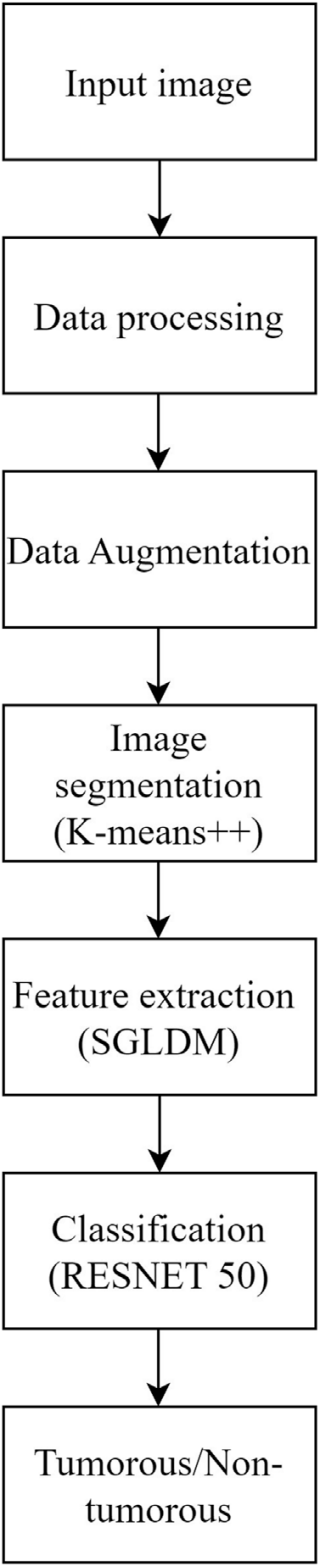
Flowchart of the proposed methodology..

The objective of this study is to address the significant challenge of accurately and reliably finding brain tumors in MRI scans, particularly at early stages where the visual differences between healthy and diseased tissues are minimal and difficult to detect. Early detection of brain tumors is crucial for improving patient outcomes, yet the subtle nature of early-stage tumors often leads to misdiagnosis or delayed diagnosis with conventional imaging techniques. The accuracy and applicability of traditional methods for brain tumor detection are limited, making it essential to develop more effective diagnostic tools. Therefore, this study focuses on creating an advanced method that can effectively differentiate between healthy and sick tissues in MRI scans, enhancing the early detection and treatment of brain tumors. The proposed methodology addresses this concern by utilizing K-means++ segmentation, SGLDM characteristic extraction, ResNet50 classification, and synthetic data augmentation to enhance the precision and adaptability of the model. This is represented through the flowchart in Figure 1. The ultimate goal is to create a tool that can assist medical professionals in precisely and promptly detecting brain tumors, resulting in better patient outcomes. This unique amalgamation sets our approach apart from existing methods and contributes to addressing the specific challenges associated with Brain Tumor detection.

The key offerings of this paper are:

• Accurate Segmentation of tumor region using K-means++ algorithm which minimizes the noise and initialization sensitivity.
• Efficient feature extraction using the Spatial gray level dependence matrix (SGLDM) to capture spatial correlations between pixels in an image, resulting in a more comprehensive representation of tumor regions.
• Robust classification of tumorous and non-tumorous Brain MR scans using ResNet50, results in faster training and better accuracy. An attribute-based technique, Grad-CAM was employed for better interpretability of the tumorous scans.
• The image dataset is subjected to synthetic data augmentation methods, that boosts the volume of training data and enhance the effectiveness of the deep learning model without overfitting.
• The findings reveal that the suggested approach performs better than current approaches in terms of sensitivity and accuracy, highlighting its potential for enhancing brain tumor detection and identification.

This paper is sectioned as follows. Section 2 will detail the suggested strategy for the detection of brain tumors, encompassing the procedures for data augmentation and model training. This section will provide an overview of the methodology used for detection along with the steps taken to train the model. In Section 3, the experimental results will be presented, and a comparison will be drawn between the proposed method and the existing state-of-the-art methods. This section will focus on the findings of the research and how they compare to previous research in the field. Finally, in Section 4, the paper will be concluded, and the implications of the suggested approach will be discussed. This section will summarize the main points of the paper and examine the potential impact of the research on the medical field.

## 2 Methods

### 2.1 Dataset

The data used was gathered from Kaggle, a website that offers publicly accessible datasets for data analysis and machine learning. The dataset utilized is Kaggle’s Br35H::Brain Tumor Detection 2020 dataset (available at Br35H:: Brain Tumor Detection 2020 (kaggle.com)), which includes 3,060 images of both tumorous and non-tumorous brain MRI scans. Out of these, 802 images—401 from each category—were chosen to create a new dataset. Our research makes use of this new dataset. We selected this dataset because it has a high number of images, which can aid in improving the precision of our model, and because it has both images with and without brain tumors, which are necessary for training a binary classification model. We chose MRI images for our study because MRI is the most effective technology for detecting brain malignancies.

### 2.2 Implementation platform

The proposed methodology is implemented and tested on the specified dataset using Google Colab, a cloud-based platform that supports Python 3.9.16. The computations are performed on a laptop equipped with a 12th Gen Intel(R) Core(TM) i5-1235U processor, operating at 1,300 MHz, featuring 10 cores and 12 logical processors. The operating system used is Microsoft Windows 11.

### 2.3 Data pre-processing

Before we could train our model, we needed to pre-process the data to prepare it for use in the deep learning model. Resizing the images from their initial dimensions to the necessary input size of 224 × 224 pixels constitutes the initial phase in pre-processing raw data for the deep learning model. To train the deep learning model, all Images must comply with the same size and aspect ratio. Upon scaling, the image’s pixel values are normalized to scale between 0 and 1. This is commonly accomplished by multiplying each pixel value by the highest potential value of the image, which is 255 in the case of an 8-bit image. The model learns more quickly and effectively when the pixel values are normalized. It also stops the gradients from growing too wide, which can be problematic during training.

**FIGURE 2:**
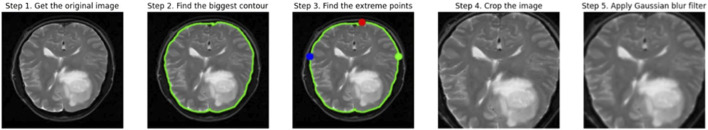
Image Pre-processing.

The images are then subjected to a Gaussian blur filter in order to eliminate any potential noise and artifacts. A common method for smoothing out images by removing high-frequency elements like noise and edges is the Gaussian blur filter. It operates by convoluting the image using a bell-shaped-curve function called a Gaussian kernel. The standard deviation of the Gaussian kernel, which is normally set to a low value to prevent blurring significant features in the image, regulates the amount of smoothing. Figure 2 shows the visual representation of the data pre-processing technique.

### 2.4 Data augmentation

**FIGURE 3:**
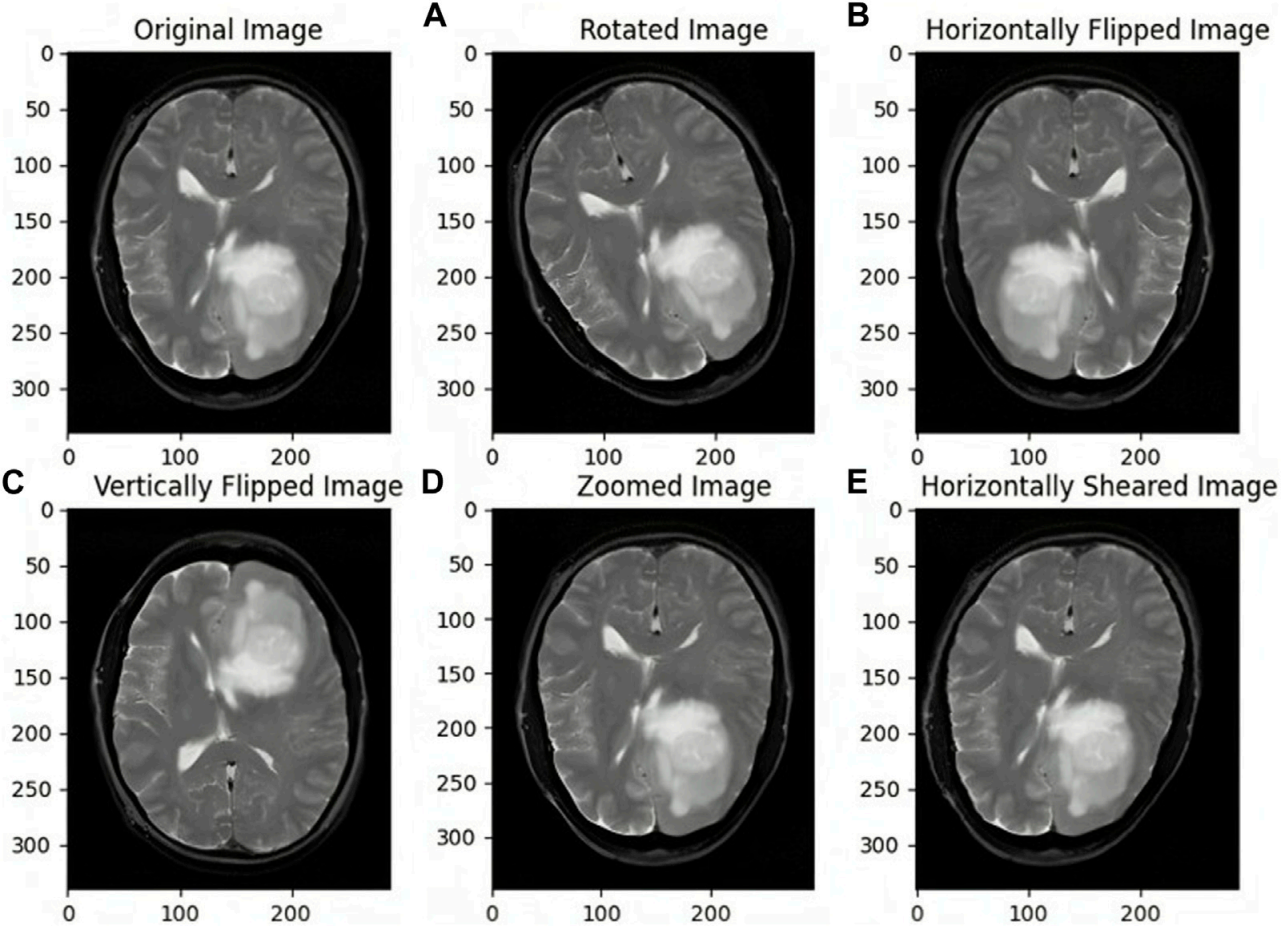
Images before and after Data Augmentation representing the original image **(A)** Rotated image **(B)** Horizontally Flipped image **(C)** Vertically Flipped image **(D)** Zoomed image **(E)** Horizontally Sheared image.

Synthetic data augmentation is employed to generate additional modifications of segmented MRI images, such as rotations, translations, and alterations in brightness and contrast, to enhance diversity and robustness of the training dataset. These random transformations help the model generalize better, reducing the risk of overfitting and improving its performance. Specifically, fundamental augmentation techniques such as rotation, shifting, shearing, zooming, flipping, and fill mode, which manages how vacant pixels are filled after transformations, were used. This process is crucial in medical imaging, where tumors can appear in various forms and orientations, and adjusting brightness and contrast mimics the variability in MRI scans. Consequently, the dataset size was expanded from 802 to 1,604 images, providing a more comprehensive training set. As illustrated in Figure 3, the augmented images demonstrate the effectiveness of these transformations in creating a robust training dataset. This approach is supported by studies like those by Shorten and Khoshgoftaar (2019), which highlight the significant improvements in deep learning model performance and generalization through data augmentation techniques.

### 2.5 Image segmentation

To segment the brain tumors from MR images, K-means++ clustering technique was employed. This is a well-known clustering technique that distributes data points to a predetermined number of clusters according to how similar they are. The initial centroid of each cluster is chosen at random by the algorithm, which then iteratively updates the centroid by calculating the mean of all the clustered data points. After the centroids stop moving appreciably or when the allotted number of iterations has been achieved, the algorithm stops.

The K-means++ algorithm delivers more accurate centroid initialization. This may contribute to better segmentation accuracy by ensuring that the clusters created throughout segmentation are more indicative of the underlying distribution of the data. The initial centroids are more uniformly spread among the data points in K-means++, making it less sensitive to the original beginning points and decreasing the likelihood of becoming stuck in an unfavorable local minimum.

Due to the initial centroids being chosen in a way that promotes the clusters being well-separated, this can converge more quickly. As a result, fewer rounds may be necessary to obtain convergence. Because the initial centroids are less likely to be impacted by extreme data points, the clustering results are more stable, making them more resistant to outliers and noise.

Functioning of the K-means++ algorithm

• The procedure begins by choosing the initial cluster centroid from a random data point.
• Based on how far they are from the current cluster centroids, the remaining centroids are selected. The likelihood that a data. In our study, we used the Euclidean distance metric to find the subsequent cluster of centroids. It is calculated as shown in Eq. 1.

$$d\;\left(a,b\right)=\sqrt{\sum_{i=1}^{q}{\left(a_{i}-b_{i}\right)}^{2}}$$ (1)

Where a, and b are two points in Euclidean “q-space”, ai and bi are Euclidean vectors, starting from the origin of the space and q refers to q-space

• After selecting all the centroids, the algorithm places each data point on the closest centroid.
• The centroids are then recalculated by the algorithm using the new data points.
• Steps 4 and 5 are repeated till the centroids stop moving noticeably or up to a predetermined number of repetitions.

Brain MRI scans can be automatically segmented into regions that are likely to represent the tumor and those that represent healthy brain tissue using the K-means++ algorithm. As a result, medical professionals may be better able to pinpoint the location of the tumor and gauge its extent for diagnostic and therapeutic purposes. In comparison to many other segmentation techniques, utilizing K-means++ for brain tumor segmentation can produce findings that are more precise, reliable, and effective.

**FIGURE 4:**
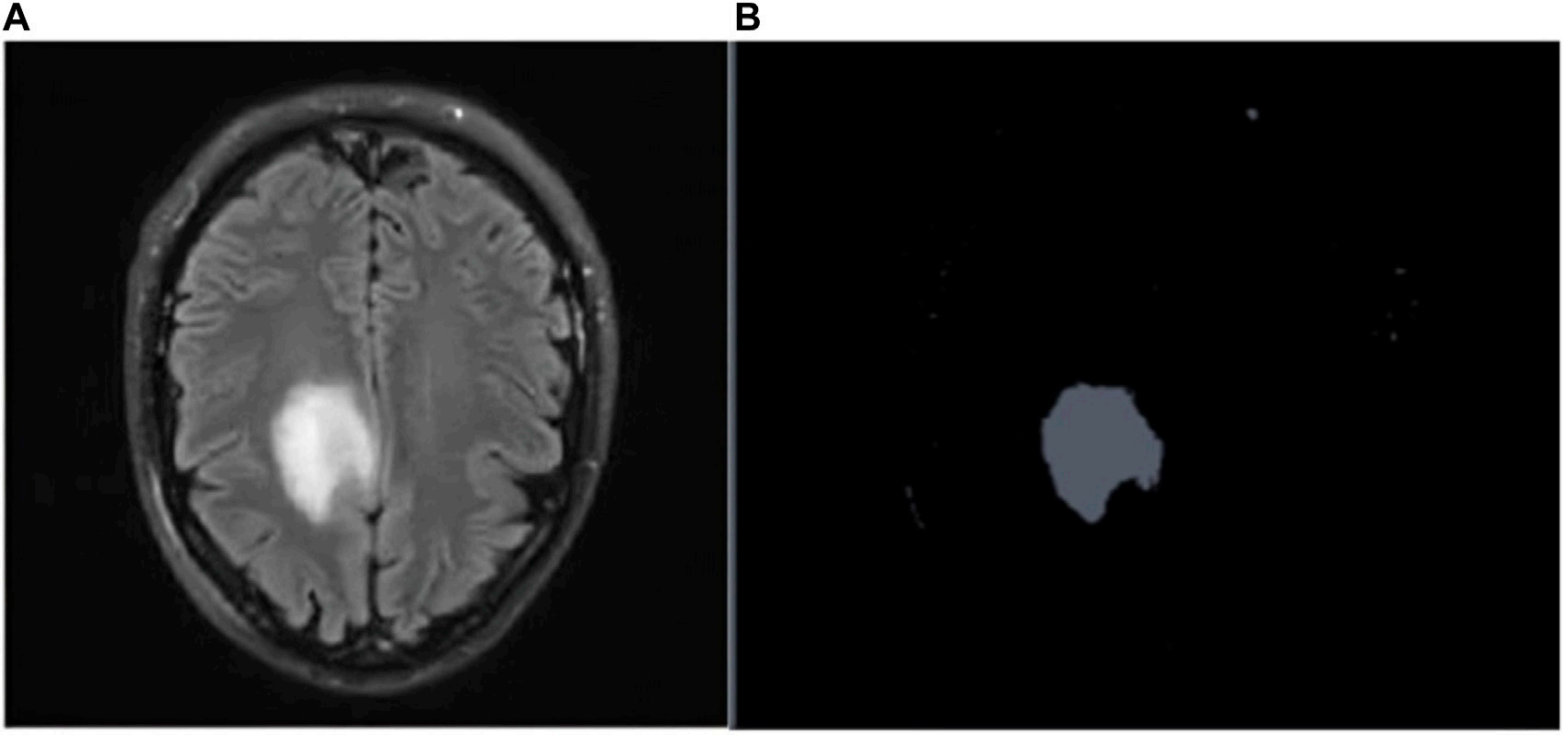
**(A)** Raw MR scan of brain tumor **(B)** Tumor after segmentation.

Figures 4A, B depicts the Raw MR image of a Brain tumor, which is then segmented using K-means++.

### 2.6 Feature extraction

In our study, textural features were extracted using the Spatial gray level dependence matrix (SGLDM) as a feature extraction method. Based on the gray-level values of the pixels in an image, SGLDM calculates the spatial dependencies between them. A gray-level dependence matrix (GLDM), which is produced by this method, measures the distribution of pairs of pixels with particular gray-level values and particular spatial relationships.

The SGLDM represents a distribution in two dimensions that relies on the frequency of traversing from 1 Gy level i to another gray level j. This is achieved by taking into account the inter-sample distance D and the direction angle θ. Consider two pixels f(X1, Y1) and f(X2, Y2) positioned at coordinates (X1, Y1) and (X2, Y2) within an image, separated by a distance d along the horizontal axis. The resulting SGLDM value P (i, j | D, θ) is thus derived.

It is represented as shown in Eq. 2.

$$P\left(i,j\right)\equiv P\left(i,j|D,\theta \right)=*\;\left\{(\left(X1,Y1\right),\left(X2,Y2\right)\;\epsilon \;W|D=\left(X1\;–\;X2,Y1\;–\;Y2\right),\left(X1,Y1\right)=i,f\left(X2.\;Y2\right)=j,\theta \right\}$$ (2)

Where * represents the frequency of appearances, (X1-X2, Y1-Y2) indicates the dot product of (x1-x2) and(Y1-Y2). The image domain, denoted as W, is specified as in Eq. 3.

$$W=\{\left(x,y\right)\left.\;\right|x\epsilon \left[0,Nx\right],y\epsilon \left[0,Ny\right]\;;\;x\;and\;y\;are\;integers$$ (3)

Here, N refers to the largest dimension of the image in both horizontal and vertical directions.

Steps involved in the calculation of SGLDM features:

• Select a tumor-containing ROI in the image, typically the output of segmentation.
• Convert the image to gray scale.
• Set the number of gray scale levels (usually 16 or 32).
• Using a specified distance and direction, compute the co-occurrence matrix (GLCM) for the selected ROI.
• Normalize the co-occurrence matrix by dividing each member by the sum of all entries in the matrix.
• Calculate the sum of each diagonal and off-diagonal element for each gray level to get the SGLDM from the normalized GLCM.
• Calculate SGLDM properties like contrast, homogeneity, and energy.
• Apply these features as input to a classifier, such as ResNet50 in our case, for tumor classification.

We specifically estimated the energy, dissimilarity, correlation, homogeneity, and contrast statistical aspects from the SGLDM.

Dissimilarity: Dissimilarity is a metric used to determine how dissimilar two things or data points are from one another as calculated in Eq. 4.

$$K=\sum_{i,j=1}^{N-1}P_{i,j}\left|i-j\right|$$ (4)

Energy: The energy refers to the sum of the squared gray level values which is calculated as in Eq. 5.

$$E=\sum_{i,j=0}^{N-1}〖P_{i,j}\left(-\ln P_{i,j}〗\right)$$ (5)

Homogeneity: It gauges how similar adjacent pixels are to one another and it is calculated using Eq. 6.

$$H=\sum_{i,j=0}^{N-1}\frac{P_{i,j}}{1+{\left(i-j\right)}^{2}}$$ (6)

Contrast: Contrast is a measurement of the variations in intensity levels between adjacent pixels, calculated using Eq. 7.

$$C=\sum_{i,j=0}^{N-1}P_{i,j}{\left(i-j\right)}^{2}$$ (7)

Correlation: The degree to which two parameters are linearly related to one another is measured by correlation as in Eq. 8.

$$L=\sum_{i,j=0}^{N-1}P_{i,j}\left[\frac{\left(i-\mu_{j}\right)}{〖{\left(\sigma 〗\right.}_{i}^{2})\left(\sigma_{j}^{2}\right)}\;\right]$$ (8)

The SGLDM features were taken from the segmented MRI images and fed into the classification deep learning model. We choose SGLDM as a feature extraction method since it has been demonstrated to be successful in extracting texture characteristics from medical Images and has been applied in numerous studies for the identification of brain tumors. The capacity for the detection of brain tumors in MRI images may be enhanced overall by combining SGLDM features with additional image features and deep learning algorithms.

### 2.7 Classification

For image classification tasks, a convolutional neural network design called ResNet50 is frequently employed. In our study, we classified brain MRI images as either having tumors or not using the ResNet50 model. Utilizing the use of transfer learning an improvised ResNet50 model is developed, which had already been trained using the Image net dataset. Convolutional layers, pooling layers, and fully connected layers are among the 50 layers that make up the ResNet50 model. The fully connected layers are in charge of classifying the images, while the convolutional layers are in charge of identifying features in the input images. The pooling layers contribute to the model’s increased efficiency by reducing the spatial dimension of the feature maps produced by the convolutional layers.

**FIGURE 5:**
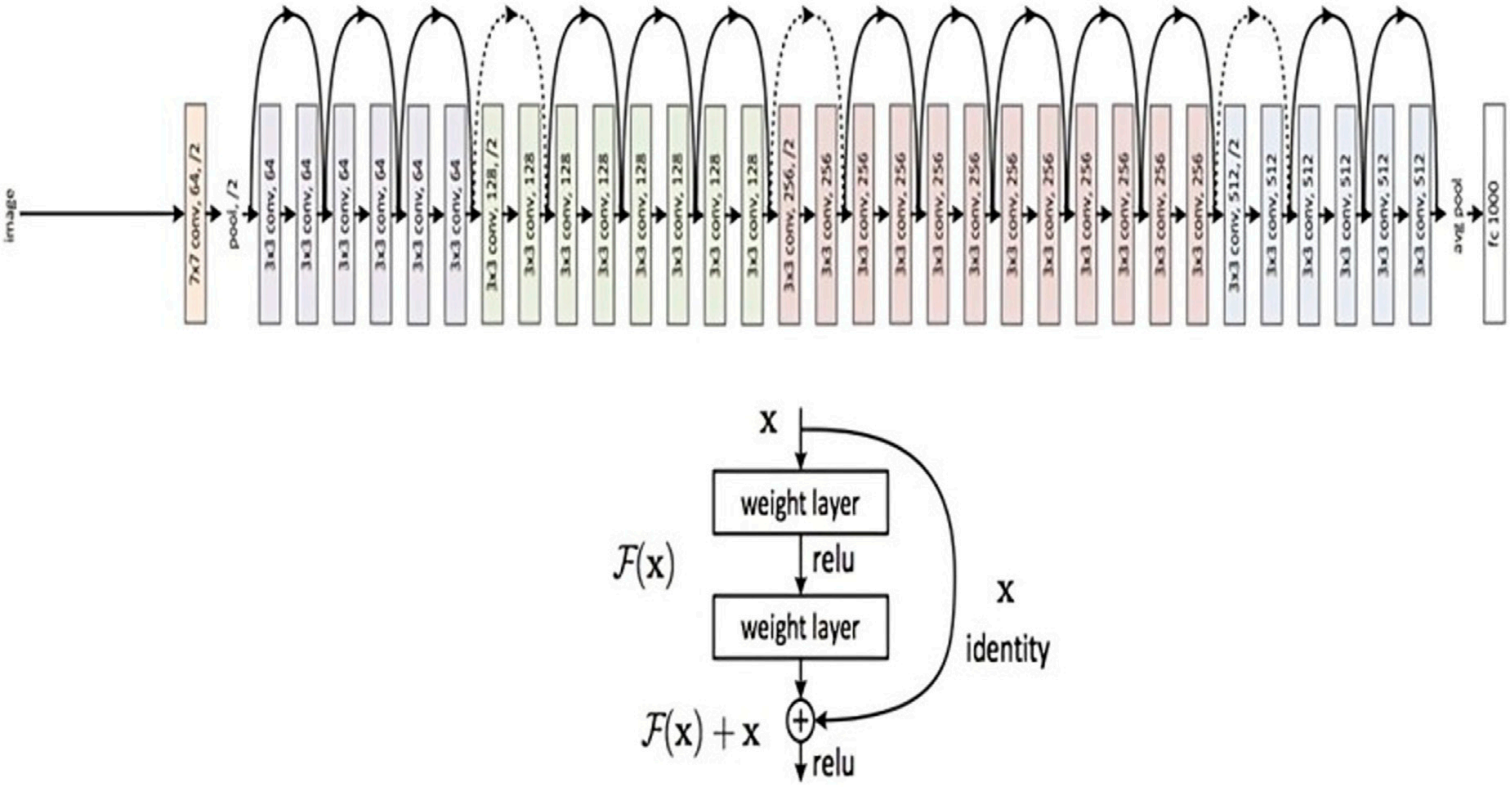
ResNet 50 Architecture Overview (reprinted from Jananisbabu, 2024).

Figure 5 presents a concise representation of the ResNet50 architecture, a key element in the experimental framework of our study. The ResNet50 model was first placed onto the ImageNet dataset with pre-trained weights, and all of its layers were frozen to stop further training. On top of the pre-trained model, two fully connected layers were added, of which, the first one had 512 neurons and a Rectified Linear Unit (ReLU) activation function, and the latter of which had a single neuron and a sigmoid activation function for binary classification. The loss function used was binary cross-entropy.

A ReLU activation function was employed in every convolutional layer. Through the activation function, the input-weighted sum is converted into the output of the node. ReLU is mathematically represented by Eq. 9.

$$f\left(W\right)=\max\left(0,W\right)$$ (9)

‘W' represents the input in this case. When W is negative or equal to zero, the negative input is transformed to zero. Inputs greater than zero yield a value of 1. As a result, in the ReLU function, the neuron is considered dead and is not triggered if the input is 0.', which is shown in Eq. 10.

$$f\;\left(w\right)=\begin{array}{c} 1,for\;w\geq 0\\ 0,for\;w\;<\;0\; \end{array}$$ (10)

In deep learning, the loss function is used to compute the variance amid the actual label values and the values predicted algorithmically. Thereafter, any optimization technique is employed to reduce the error to its lowest potential value. For the classification of MRI images as binary in nature, we utilized binary cross entropy. In binary calculations, the cross-entropy error rate varies between 0 and 1, and is represented mathematically as such in Eq. 11.

$$J\left(w\right)=w\log K\left(w\right)+\left(1\;–\;w\right)\log\left(1–\;k\left(w\right)\right)$$ (11)

The predicted label in this instance is k(w), and the actual label is w. Because w is multiplied by the log, when the actual labels, w, are equal to 0, the first term will be zero. Similarly, the second constituent also becomes zero, When w = 1. If w = k(w), N(w) will be 0.

Various optimization methods are available to diminish the loss in deep neural networks by altering parameters such as weights and learning rates. In our investigation, we utilized the Adaptive Moment Estimation (Adam) optimizer, which combines momentum-based stochastic gradient descent with RMSprop.

During every epoch of stochastic gradient descent, we calculate the weight (dN) and bias (dM) derivatives, which are then multiplied by the learning rate, as in Eq. 12.

N=N – η×dNM=M – η×dM (12)

For the current batch, we computed dN and dM, and the results were a moving mean between 0 and 1. The moving mean of the gradients is obtained using stochastic gradient descent with momentum I, calculated as in Eq. 13.

$$\mathrm{IdN}=\gamma \times \mathrm{IdN}+\left(1\;–\;\gamma \right)\times \mathrm{dNIdM}=\gamma \times \mathrm{IdM}+\left(1\;–\;\gamma \right)\times \text{dM}$$ (13)

Similar to this, Hinton developed the Root Mean Squared Prop as an adaptable learning rate technique [46]. We employ RMSProp’s exponential moving mean square of the gradients. The mathematical representation of RMSProp is given in Eq. 14,

$$RdN=\gamma \times RdN+\left(1\;–\;\gamma \right)\times dN2RdM=\gamma \times RdM+\left(1\;–\;\eta \right)\times dM2$$ (14)

The exponentially weighted means are adjusted using a hyperparameter known as Gama γ. The traits of the weighted mean and the weighted mean of the squares of the prior gradients were combined to employ the Adam optimization strategy. Therefore, the revised weights and bias of the Adam Optimizer will be as in Eq. 15.

$$N=N\;–\;\eta \times \left(I_{{dN}^{/}}\sqrt{R_{dN}}+R\right)$$

$$M=M\;–\;\eta \times \left(I_{{dM}^{/}}\sqrt{R_{dM}}+R\right)$$ (15)

Zero division is eliminated by Epsilon R (Epsilon = 10-8), and η stands for the learning rate.

The output of the feature extraction phase was deployed as the input to the ResNet50 model. The categorical cross-entropy loss function was utilized to determine the variance between the predicted and actual labels during the training process. The model was trained for 30 iterations with the Adam optimizer and a learning rate of 0.001.

**FIGURE 6:**
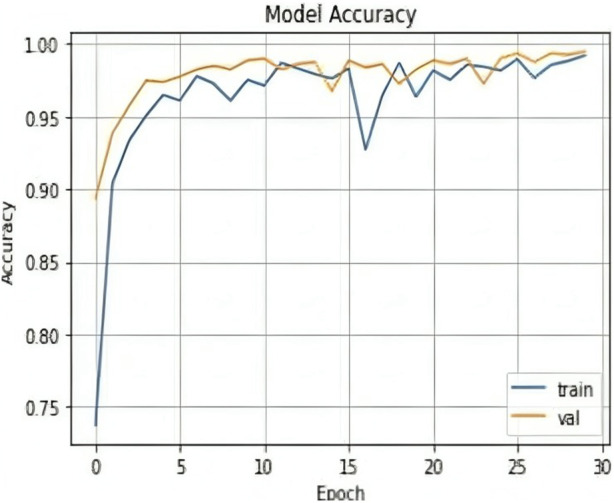
Accuracy graph of the proposed model.

Additionally, the inclusion of Gradient-weighted Class Activation Mapping (Grad-CAM) has been done, following the convolutional layers of our ResNet50 architecture, to enhance the interpretability of our brain tumor classification model. Grad-CAM is a visualization method that helps us identify the areas of the brain MRI scans that have a major impact on the final categorization. Grad-CAM computes the gradients of the anticipated class scores with respect to the feature maps after the convolutional layers have extracted features. The spatial areas that contribute most to the classification output are highlighted in a heatmap created using these gradients. We may learn a great deal about the characteristics and regions that the model gave priority to throughout the classification process by superimposing these heatmaps on the original MRI images. In addition to offering a potent tool for model interpretation, this inclusion contributes to the development of trust and comprehension in the clinical application of our approach for classifying brain tumors. Examples of the Grad-CAM heatmaps that highlight the regions of interest found by our model are shown in Figure 6.

Following training, we assessed the model performance of ResNet-50, using the performance measures mentioned below on the validation set. A confusion matrix was also used to visualize how well the model performed.

## 3 Experimental results and discussion

### 3.1 Experimental setup

The proposed methodology is tested on the aforementioned dataset using Google colab, which uses Python 3.9.16, on a laptop with a Processor 12th Gen Intel(R) Core (TM) i5-1235U, 1,300 MHz, 10 Core(s), 12 Logical Processor(s), and Microsoft Windows 11.

The initial dataset was split into training and testing data in a 70:30 proportion.

### 3.2 Performance metrics

The effectiveness of our suggested method was assessed using the subsequent performance metrics, as in Eqs 16–19 respectively.

Accuracy: The percentage of correctly categorized images in the test set as compared to all the images in the test set.

$$Accuracy=\frac{TP}{Total\;number\;of\;predictions}$$ (16)

Precision: The ratio of correctly predicted positives (TP) to all predicted positives (TP + FP), where TP is the count of accurate positive predictions and FP is the count of inaccurate positive predictions.

$$Precision=\frac{TP}{TP+FN}$$ (17)

Recall: The ratio of correctly predicted positives (TP) to all actual positives (TP + FN), where TP represents the count of accurate positive predictions and FN represents the count of inaccurate negative predictions.

$$Recall=\frac{TP}{TP+FN}$$ (18)

F1 score: This is a single score that balances both metrics and is computed as the harmonic mean of precision and recall.

$$F1\;score=2*\frac{precision*recall}{Precision+recall}$$ (19)

### 3.3 Results and discussions

#### 3.3.1 Performance of the proposed model

Table 1 shows the features that are extracted from the tumorous and non-tumorous MR images.

**TABLE 1:** Features extracted from random images in the dataset using the SGLDM method.

Image number	Contrast	Dissimilarity	Homogeneity	Energy	Correlation
Y32 [Tumorous Image]	696.974707	1,136.726091	502.257311	1,024.496	11.57009
Y313 [Tumorous Image]	504.299777	829.300708	387.387861	706.5730	10.46417
Y390 [Tumorous Image]	2487.90452	3,673.003989	2129.828718	3,817.196	28.23669
Y397 [Tumorous Image]	1737.46131	2608.654610	1,451.894017	2751.701	22.64054
Y398 [Tumorous Image]	1,226.44633	1890.735612	932.658447	1920.585	17.11975
No6 [Non-Tumorous Image]	106.580234	172.769611	77.178417	170.1018	5.080357
No50 [Non-Tumorous Image]	143.365402	222.801614	102.2258832	216.1244	6.358559
No56 [Non-Tumorous Image]	60.419799	96.324885	42.411867	100.1154	2.982598
No364 [Non-Tumorous Image]	168.464468	218.7196	94.853518	233.4348	6.268135
No396 [Non-Tumorous Image]	54.576343	78.760849	24.918817	76.10711	2.132773

The results shown in Table 2 demonstrate that the suggested model attained remarkable levels of accuracy throughout the training and validation periods. The model specifically had a training accuracy of 99.25%, meaning it could correctly predict the class for 99.25% of the training data. Also, the validation accuracy increased to 99.50%, indicating that the model performed well when applied to fresh, untested data. Also, the training loss was 0.024, indicating that throughout training, the model’s predictions were reasonably accurate.

**TABLE 2:** Training and validation accuracies and losses of the Proposed model.

	Training	Validation
Loss	0.024101	0.017044
Accuracy	0.992519	0.995012

**FIGURE 7:**
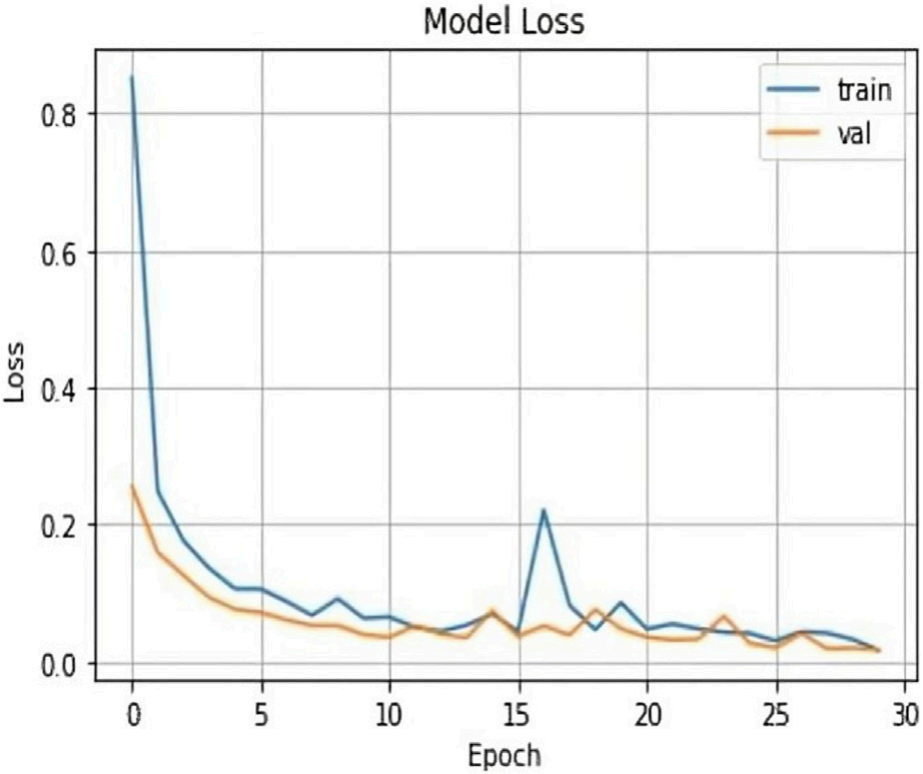
Loss graph of the proposed model.

**FIGURE 8:**
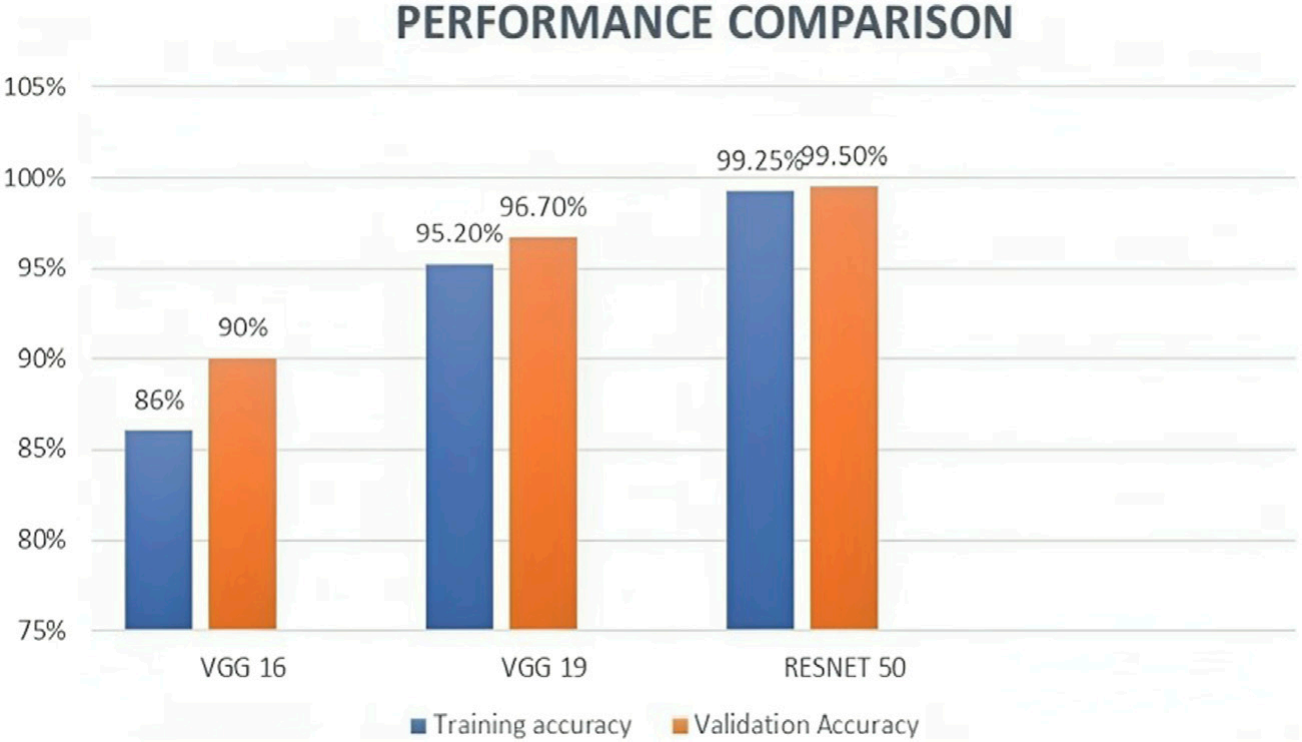
Bar graph showing the proposed model with the existing Transfer learning model.

The validation loss, which was again 0.017, showed that the model could correctly predict the classes of the validation data. Figures 7, 8 represent the Model accuracy and loss graphs respectively.

The evaluation results of the proposed model is presented in Table 3. The precision, accuracy, recall, and F1-Score of the algorithm were derived by employing the aforementioned equations. The findings imply that the suggested algorithm surpassed alternative state-of-the-art techniques.

**TABLE 3:** Classification report of the proposed model.

	Precision	Recall	F1-score	Support
0	1.00	1.00	1.00	20
1	1.00	1.00	1.00	12
Accuracy	1.00	1.00	1.00	32
Macro average	1.00	1.00	1.00	32
Weighted average	1.00	1.00	1.00	32

#### 3.3.2 Performance comparison of the proposed model with state-of-art methods

ResNet50, VGG16, and VGG19 are some of the most well-liked and frequently employed architectures for deep learning models for image classification. Deep convolutional neural network architectures like ResNet50, VGG16, and VGG19 vary in the model’s complexity and number of layers. ResNet50 features 50 layers, compared to 16 and 19 layers for VGG16 and VGG19, respectively. ResNet50 has been demonstrated to perform better than both VGG16 and VGG19 on a number of image classification tasks. This is because, in comparison to VGG16 and VGG19, it uses residual connections, which enable the network to learn and improve feature representations. In addition, the usage of skip connections in ResNet50 makes it more computationally efficient than VGG19 even though it has more layers. VGG16 and VGG19, however, each have advantages of their own. These models are simpler to train and use than ResNet50 because of their simplified architecture. Moreover, transfer learning works well with VGG16 and VGG19, enabling the models to be tailored for certain image classification tasks with fewer data. Due to their enormous capacity and capacity to learn complicated feature representations, VGG16 and VGG19 have also been demonstrated to perform well on datasets with small images or little training data.

**FIGURE 9:**
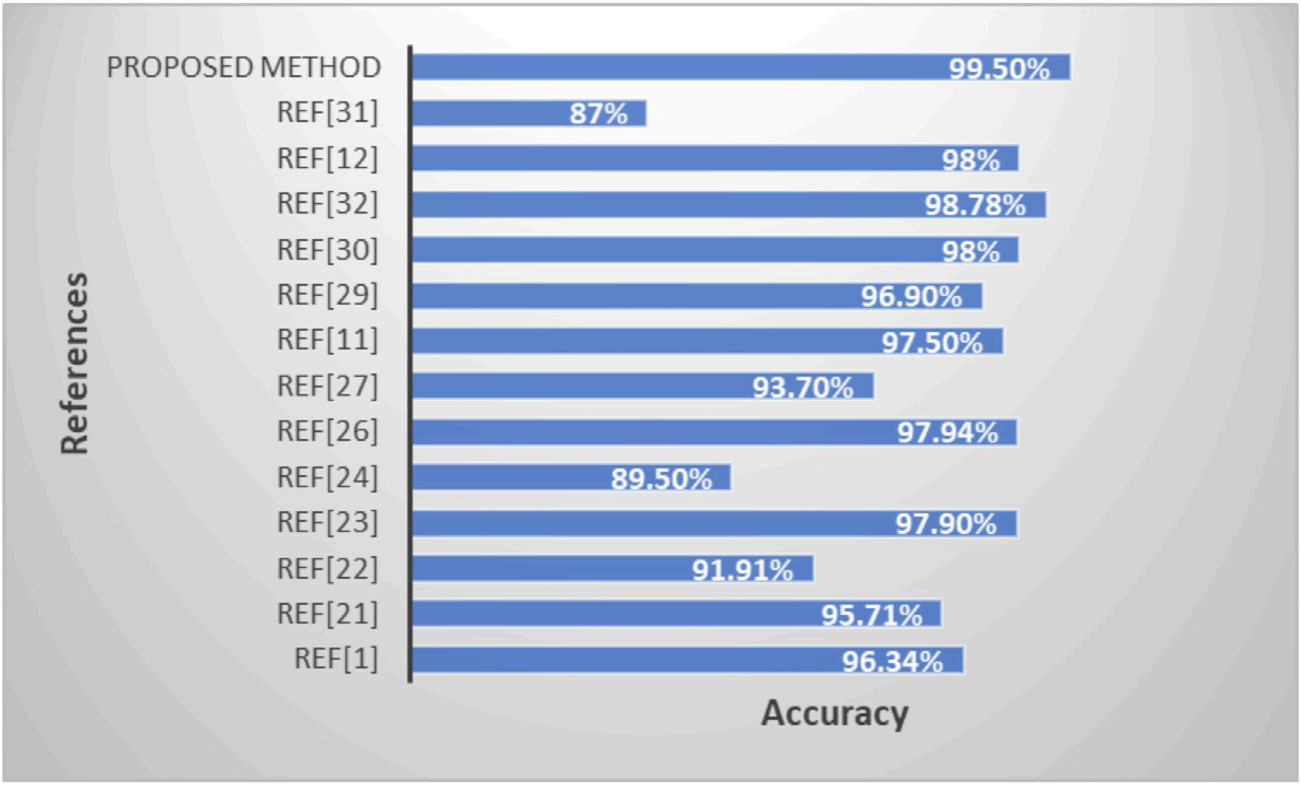
Bar graph showing the performance comparison of the proposed model with Existing models.

We also experimented VGG 16 and VGG 19 using the same dataset along with the same preprocessing, segmentation, and feature extraction methods, to compare the performance of these models with ResNet50. According to the results, ResNet50 had the best accuracy, scoring 99.50, while VGG16 and VGG19 scored 90% and 96.75%, respectively. Table 4 and Figure 9 represents the same. These findings are in line with earlier research, which demonstrated that ResNet50 outperformed VGG16 and VGG19 on a range of Image classification tasks.

**TABLE 4:** Performance Comparison of Proposed Model with other Transfer learning models.

Model	Training accuracy (%)	Validation accuracy (%)	Dataset used
VGG16	86	90	Br35H::Brain Tumor Detection 2020 dataset (Kaggle, 2020)
VGG19	95.2	96.7	Br35H::Brain Tumor Detection 2020 dataset (Kaggle, 2020)
ResNet50	99.25	99.50	Br35H::Brain Tumor Detection 2020 dataset (Kaggle, 2020)

To sum up, ResNet50, VGG16, and VGG19 are all effective deep-learning models for classifying images, each with specific advantages and disadvantages. ResNet50 is more accurate and computationally efficient than VGG16 and VGG19, yet VGG16 and VGG19 are easier to use and better suited for transfer learning.

In comparing the transfer learning approach, utilizing pre-trained ResNet50, with Graph Neural Networks (GNN), the transfer learning model excels in spatial relationship modeling. Through its convolutional layers, it adeptly captures hierarchical spatial features crucial for segmentation, providing a foundation for precise spatial understanding. Additionally, the transfer learning model seamlessly integrates contextual information within learned hierarchical features, contributing to robust tumor segmentation capabilities. Its strength in handling irregular tumor shapes, derived from adaptability during pre-training on diverse image datasets, sets it apart. Furthermore, the model demonstrates computational efficiency, making it a practical choice for medical image segmentation and classification, while GNN may encounter challenges in computational complexity for large-scale datasets. This analysis emphasizes spatial and contextual relationships, highlighting the transfer learning technique as a powerful mechanism for more successful brain tumor detection.

#### 3.3.3 Performance comparison of the proposed model with existing methods

**FIGURE 10:**
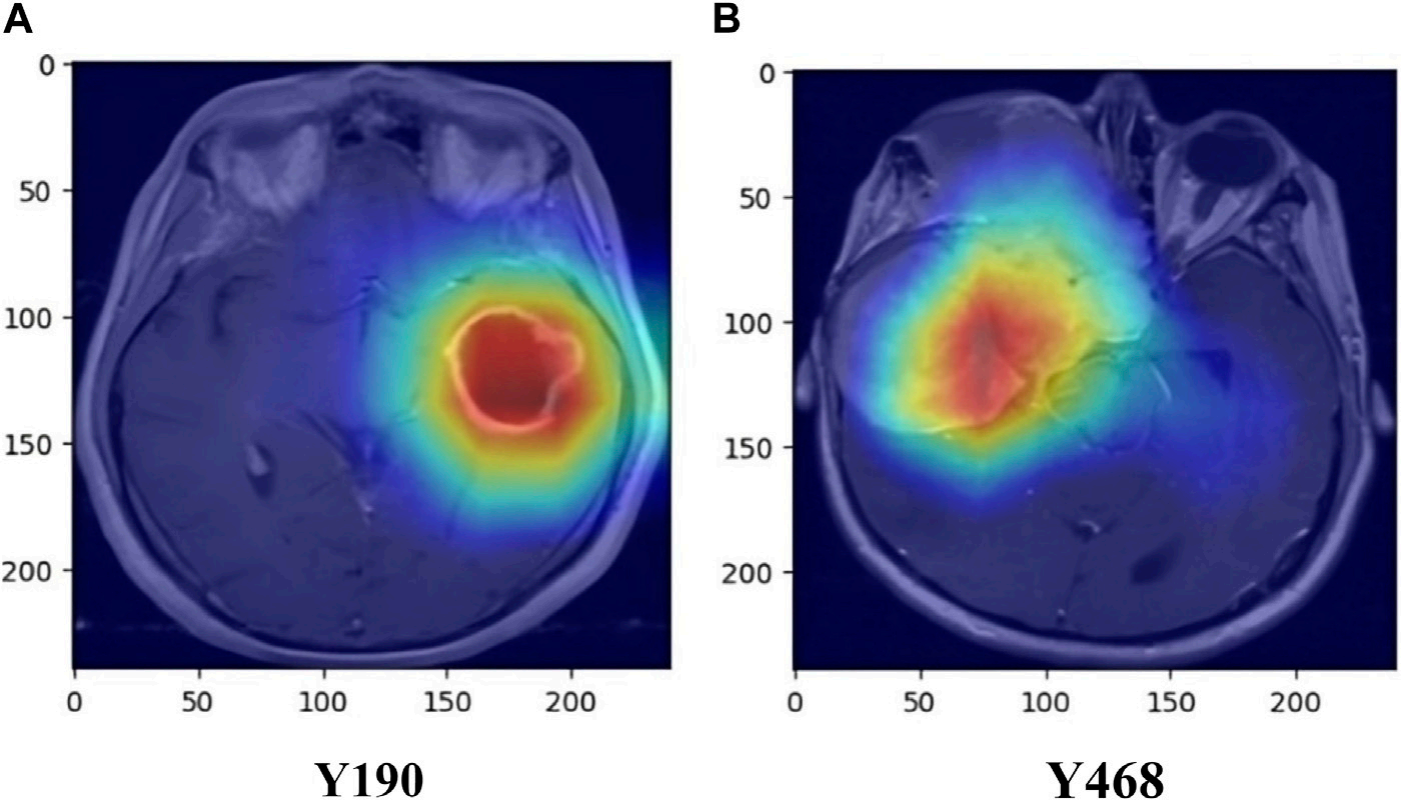
Heat map visualization of images using Grad-CAM **(A)** Y190 **(B)** Y468.

Table 5 and Figure 10 shows the performance comparison of the proposed model with existing state-of-art models.

**TABLE 5:** Performance comparison of proposed model with existing state-of-art methods.

S.NO	Authors	Methods used	Dataset used	ACCURA CY
1	T. Saba et al. (Saba et al., 2020)	wavelet-based texture features and morphological features as hand-crafted features, and a convolutional neural network	BRATS2015	96.34%
			BRATS2016	
			BRATS2017	
2	S. Deepak and P. M. Ameer (Khawaldeh et al., 2017)	VGG16-SVM	Dataset from Figshare	95.71%
3	P. Saxena et al. (Tazin et al., 2021)	CNN, LSTM	BRATS	91.91%
4	A. Çinar and M. Yildirim. (Sarah et al., 2023)	VGG16, ResNet50	BRATS	97.9%
5	S. Khawaldeh et al. Cheng et al. (2015)	CNN	The Cancer Genome Atlas (TCGA) Low Grade Glioma (LGG) collection	89.5%
6	Ö. Polat and C. Güngen. (Khan et al., 2020)	VGG 19	BRATS	97.94%
7	J. Cheng et al. (Han et al., 2019)	SVM	A collection of MR images of brain tumors with four different types of tumors	93.7%
8	Abdelaziz Ismael et al. (Shah et al., 2020)	Enhanced residual networks like ResNet and DenseNet	BRATS	97.5%
9	M.A.; Ashraf et al. (Yan et al., 2023)	VGG-19, VGG-19	BRATS	96.9%
10	Han et al. (Munir et al., 2022)	VGG-16, Alexnet	BRATS	98%
11	Nayak et al. (Salama and Shokry, 2022)	ResNet 50, MobileNet, MobileNetV2	BRATS	98.78
12	Amin et al. (Krishnapriya and Karuna, 2023a)	SVM, naïve-bay es, ensemble DT, KNN	BRATS	98%
13	Ari et al. (Amran et al., 2022)	ResNet-50 for Detection GAN for Data Augmentation	BRATS	87%
14	Proposed method	K-means++ for segmentation, Feature extraction using SGLDM, Classification using ResNet50 along with synthetic data augmentation	Br35H::Brain Tumor Detection 2020 dataset (Kaggle, 2020)	99.50%

## 4 Limitations

Few restrictions apply to the proposed approach to detect brain tumors.

1. Quality of Input Data: The success of the proposed approach heavily relies on the quality of the input data, encompassing factors such as image resolution, noise levels, and contrast. Poor image quality or unconventional imaging techniques can compromise the accuracy of segmentation and feature extraction processes, thereby affecting the reliability of the predictions made by the model. Strategies to address this limitation may involve implementing robust preprocessing techniques to enhance image quality, standardizing imaging protocols across different systems, or exploring alternative imaging modalities that offer higher resolution and contrast.
2. Computational Power Requirements: The approach demands substantial computational resources, particularly during the training phase. This high computational overhead may pose challenges for real-time processing in practical applications, where timely diagnosis is crucial. To alleviate this limitation, optimizations such as algorithmic efficiency improvements, parallel computing techniques, or utilization of specialized hardware accelerators (e.g., GPUs, TPUs) could be explored. Additionally, techniques like transfer learning or model distillation may reduce the computational burden without sacrificing performance.
3. Capturing Real-World Variability: While the approach demonstrates promising performance, there is a concern that it may not fully capture the variability present in real-world datasets. The model training data may not adequately represent the diverse range of pathologies, imaging artifacts, and patient demographics encountered in clinical practice. To address this limitation, incorporating more diverse and representative training data, augmenting the dataset with synthetically generated examples, or employing techniques like domain adaptation could enhance the ability of the model generalize to unseen variations. Although the strategy demonstrated promising results on a particular dataset, further investigations must be conducted to determine if it can be applied to other datasets or clinical settings.
4. Generalizability of Other Datasets/Clinical Settings: Despite its efficacy on a specific dataset, the generalizability of the approach to different datasets or clinical environments remains uncertain. Variations in imaging protocols, equipment, and patient populations across institutions can impact the performance of the model. Further validation studies on external datasets, spanning multiple clinical sites and populations, are essential to assess the robustness and applicability of the approach in diverse real-world scenarios. Additionally, techniques like cross-validation, external validation cohorts, and model interpretability analyses can provide insights into the model performance across different settings and aid in identifying potential sources of bias or variability.

## 5 Discussion

While our work proposes a potential method for detecting brain tumors, a few limitations are to be noted. To begin, the proposed strategy is strongly reliant on the integrity of the input data. Image resolution, noise, and contrast can all have an impact on the accuracy of the segmentation and feature extraction steps. As a result, for datasets with poor image quality or non-standard imaging techniques, the algorithm may not perform well. Secondly, the suggested method requires a lot of computing, especially during the training phase. This could be a hindrance in actual applications that demand real-time processing. Third, despite the fact that synthetic data augmentation improved the model’s performance, it may not adequately represent the unpredictability of real-world data. The employment of more diversified and complex augmentation techniques may result in further performance improvements. Finally, while the suggested strategy outperformed existing state-of-the-art methods on the Br35H::Brain tumor detection dataset, its applicability to other datasets or clinical contexts needs to be tested further.

While our current study presents a robust transfer learning-based approach for brain tumor segmentation, the ever-evolving field of medical image analysis offers opportunities for continuous improvement and innovation. In the realm of adaptive segmentation, we foresee significant potential in the integration of Reinforcement Learning (RL) strategies. Future research endeavors will delve into the exploration of how RL can dynamically optimize segmentation pathways, adapting processing steps to the unique characteristics of medical images. The envisioned research will undertake a comprehensive analysis, comparing our transfer learning-based approach to RL strategies and exploring synergies between these methodologies. RL, known for its capacity in learning from sequential decision-making processes, holds promise in guiding the segmentation process by dynamically selecting and sequencing processing steps based on varying contexts within medical images. This adaptive approach could lead to more nuanced and context-aware segmentation models.

The combination of prior knowledge encoded in transfer learning models with RL’s adaptive decision-making capabilities is expected to enhance segmentation precision, making the approach more flexible and adaptable across diverse medical imaging scenarios. This research not only aligns with current trends in medical image analysis but also sets the stage for the development of more dynamic and responsive segmentation models. The outcomes of this investigation have the potential to contribute valuable insights to the broader field of adaptive medical image analysis, paving the way for advancements that transcend specific applications.

## 6 Conclusion

This proposed work concludes with a method for detecting brain tumors that combines segmentation using K-Means++, feature extraction from SGLDM, classification using ResNet50, Visualization through Grad-CAM, and data augmentation using synthetic data. The suggested method seeks to improve the accuracy and resilience of detecting brain tumors while requiring as little manual intervention as possible. Testing on the Br35H::Brain Tumor Detection 2020 dataset revealed that the suggested method outperforms the existing state-of-the-art methods. The incorporation of synthetic data augmentation also contributed to enhancing the performance of the model. To summarize, the suggested method has the potential to increase the accuracy and reliability of brain tumor detection, which has important implications for early diagnosis and treatment. Future research could include testing the approach on larger and more varied datasets, as well as in real-world clinical situations. This research serves as a foundation for future research into more complex tumor segmentation and classification techniques.

## Data Availability

The original contributions presented in the study are included in the article/Supplementary Material, further inquiries can be directed to the corresponding author.
